# The central mediating effect of inhibitory control and negative emotion on the relationship between bullying victimization and social network site addiction in adolescents

**DOI:** 10.3389/fpsyg.2024.1520404

**Published:** 2025-04-02

**Authors:** Jiale Wang, Ning Wang, Tianci Qi, Yang Liu, Zhenhua Guo

**Affiliations:** ^1^School of Sports Science, Jishou University, Jishou, China; ^2^School of Sports Science, Guangxi Minzu University, Nanning, China

**Keywords:** bullying victimization, social network site addiction, depression, anxiety, stress, inhibitory control, adolescents

## Abstract

**Background and objectives:**

Bullying victimization is strongly associated with social network site addiction among adolescents. However, the underlying mechanisms between these variables remain unclear. This study aims to explore the psychological mechanisms linking bullying victimization to social network site addiction by examining the mediating roles of depression, anxiety, stress, and inhibitory control.

**Methods:**

A self-report survey was conducted among a sample of 1,005 adolescents in China. The survey included measures of bullying victimization, social network site addiction, depression, anxiety, stress, and inhibitory control. Descriptive and correlational analyses were performed, followed by the construction of a chain mediation model.

**Results:**

Bullying victimization was significantly positively associated with social network site addiction. However, this relationship became non-significant when negative emotional states (depression, anxiety, stress) and inhibitory control were introduced as mediating variables. Specifically, bullying victimization significantly predicted negative emotional states and was negatively associated with inhibitory control. Negative emotional states were also negatively related to inhibitory control, while inhibitory control was negatively associated with social network site addiction. Among the negative emotional states, stress and anxiety showed a significant negative correlation with social network site addiction.

**Conclusion:**

This study sheds further light on the psychological mechanisms linking bullying victimization and social network site addiction among adolescents. Depression, anxiety, stress, and inhibitory control act as mediating factors in this relationship. These findings highlight the importance of targeting these variables in understanding and developing interventions for social network site addiction among adolescents.

## 1 Introduction

With the rapid advancement of smart devices and social media, social network sites have become a central part of daily life, serving as important platforms for acquiring information and enhancing social interactions (Al-Khani et al., 2021). Recent statistics estimate that 62.3% of the global population uses social network sites, with an average daily usage time of 2 h and 23 min (Dave, 2024). While the widespread use of social networks brings convenience, spending excessive time on these platforms can lead to negative outcomes, such as dependence and addiction (Caner et al., 2022). In China, the internet penetration rate among adolescents is 98.5, and 95.9% of adolescents use social network sites (Park and Choi, 2022; Dave, 2024). During adolescence, forming social relationships is a key developmental task, and using social networks actively can help foster close interpersonal connections (Boer et al., 2020; Erikson, 1950). However, if social network use is not moderate, adolescents are more susceptible to social network site addiction compared to other age groups (Teng et al., 2021). According to the addiction syndrome model (Shaffer et al., 2004), social network site addiction is a complex and multifaceted phenomenon. It is thought to be triggered by distal factors (such as psychosocial issues and underlying vulnerabilities), which, through proximal factors (such as negative events), lead to excessive social network use. This behavior is eventually reinforced, intensifying the addiction (Andreassen and Pallesen, 2014). Social network site addiction, as a major form of internet addiction, is defined as an excessive preoccupation with social networks, driven by a strong urge to log in or use them, leading to significant time investment that impairs other social activities, academic performance, work, interpersonal relationships, and mental health (Andreassen and Pallesen, 2014). The diagnostic criteria for social network site addiction include preoccupation (compulsive or persistent thoughts about social networking), tolerance (increased use needed to attain satisfaction), withdrawal symptoms (unpleasant feelings when use is abruptly stopped), failure to control usage, escape (using social networks to cope with negative emotions), problems (continuing social network use despite negative consequences), deception (concealing the time spent on social networks), displacement (neglecting other activities due to social network use), and conflict (experiencing interpersonal conflicts because of excessive use) (van den Eijnden et al., 2016). This addiction leads to excessive, compulsive use of social network platforms, interfering with daily life and causing negative impacts on physical, social, and mental health (Andreassen and Pallesen, 2014). In recent years, social network site addiction has become an emerging issue among adolescents worldwide, necessitating urgent investigation into the factors associated with it to provide prevention and intervention strategies.

Among the factors contributing to social network site addiction, bullying victimization has shown a close relationship (Craig et al., 2020; Cui and Yip, 2024; Fang et al., 2022). Researchers widely view social network site addiction as a behavioral response to prior stress-inducing life events and adversities (Stockdale and Coyne, 2020; Zsido et al., 2020). Bullying victimization, one of the most common and concerning adversities faced by adolescents, places victims at higher risk for physical, cognitive, and psychological health problems (Moore et al., 2017), particularly in terms of increased rates of anxiety, depression, and suicide (Moore et al., 2018). Bullying victimization is defined as repeated and frequent negative behavior by another person in a situation of unequal power or status, encompassing both offline bullying (e.g., physical or verbal aggression) and cyberbullying (e.g., harassment or exclusion through digital platforms) (Olweus, 1993; Smith et al., 2021). Previous studies have shown that bullying victimization—whether offline or online—can result in significant emotional distress, including depression and anxiety, which may lead adolescents to turn to social networks as a coping mechanism (Gothwal et al., 2013; Gladden et al., 2014). A meta-analysis revealed a 36% prevalence of bullying victimization among adolescents, with 13.13% of Chinese adolescents reporting experiences of being bullied (Modecki et al., 2014; Ran et al., 2020). Recent studies indicate that 36% of girls and 24% of boys frequently suffer from bullying victimization (Przybylski and Bowes, 2017). Adolescents who experience bullying tend to have weaker social bonds, spend more time online, and are more likely to establish online relationships for a sense of belonging, which increases their risk of social network site addiction (Jia et al., 2018). When adolescents face peer bullying in real life, they may turn to social networks to satisfy their psychological needs and regain confidence in the virtual world, thus coping with the negative effects of bullying (Li et al., 2019). The experiences of bullying victimization during adolescence may teach individuals maladaptive behavior patterns and coping mechanisms (Waasdorp and Bradshaw, 2011), leading them to respond to such adversity by using social networks to maintain social status or avoid the stigma of victimization (Oransky and Marecek, 2009). However, research highlights that while these platforms may serve as temporary spaces for alleviating negative emotions, their overuse can foster maladaptive coping mechanisms and increase the risk of addiction (Wadsley et al., 2022; Wegmann et al., 2022). Given the review above, this study hypothesizes that bullying victimization significantly predicts the occurrence of social network site addiction among adolescents.

Depression, anxiety, and stress may serve as important mediators in the relationship between bullying victimization and adolescents’ social network site addiction. Depression, anxiety, and stress are major global mental health concerns, significantly contributing to disability worldwide and affecting people of all ages (Kessler et al., 2005). According to the World Health Organization (WHO), approximately one in four people will experience a mental health issue during their lifetime, with about 450 million individuals worldwide currently living with such conditions (Saraceno, 2002). Among adolescents, mental health issues account for 16% of the global disease and injury burden, with approximately 20% of adolescents experiencing mental health problems (Blakemore, 2019). Severe mental health issues can disrupt emotional, cognitive, and social functioning, resulting in adverse outcomes, including social network site addiction (Alavi et al., 2011). Depression, a critical public health concern, is characterized by symptoms such as lack of vitality or sadness, and up to 34% of adolescents are at risk of clinical depression (Clark et al., 2012; Shorey et al., 2022). Many studies have demonstrated a positive correlation between bullying victimization and depression, identifying bullying victimization as a significant risk factor for adolescent depression (Zhang et al., 2020; Xiong et al., 2023; Liu et al., 2024a,b,c,d,e). Anxiety, a high-risk psychological disorder in adolescents, manifests as excessive fear or worry in specific situations (e.g., panic attacks or social scenarios) and symptoms such as difficulty concentrating or making decisions (Narmandakh et al., 2021). Anxiety also increases the risk of substance use disorders (Alonso et al., 2018). Research has shown a significant positive correlation between bullying victimization and anxiety, with both victims and perpetrators of bullying exhibiting heightened anxiety risks (Gong et al., 2022; Lee, 2021). Adolescents are increasingly exposed to unhealthy levels of stress due to academic demands, family expectations, interpersonal conflicts, self-identity challenges, and future employment pressures, exacerbated by intense societal competition (Núñez-Regueiro and Núñez-Regueiro, 2021). Bullying victimization, as a chronic stressor, can inflict severe psychological trauma on adolescents (Xu et al., 2023). Studies have found a positive correlation between bullying victimization and stress, demonstrating that victimization amplifies adolescents’ psychosocial stress responses (Chen et al., 2018). According to the social information processing theory, internalizing bullying experiences may lead adolescents to develop negative self-evaluations, which, in turn, contribute to mental health problems (Wu et al., 2018). Supporting this theory, research has shown that bullying victimization can intensify negative self-perceptions and heighten feelings of insecurity and threat within one’s environment (Fabris et al., 2021). Additionally, the emotion regulation theory posits that bullying victimization may evoke intense negative emotions, impairing adolescents’ ability to effectively regulate emotional responses and leading to internalizing problems such as depression, anxiety, and stress (Dyar et al., 2024). Furthermore, growing evidence underscores the role of these negative emotions in the development of social network site addiction (Foroughi et al., 2021; Li et al., 2019; Liang et al., 2016; Nie et al., 2017; Zhao et al., 2023; Liu et al., 2024a,b,c,d,e). According to the mood enhancement hypothesis (Bryant and Zillmann, 1984), individuals experiencing unpleasant emotions are more likely to engage in leisure activities, including social networking, as a means of stress relief. Adolescents may use social networks to alleviate emotional symptoms, spending increasing amounts of time on these platforms for entertainment and relaxation (Nelson et al., 2020). Adolescents with depression, anxiety, and stress, despite desiring social interaction to meet emotional and social needs, may avoid face-to-face interactions due to fears of negative evaluation (Kashdan et al., 2008). Social networks, in contrast, provide a relatively safe space where threatening stimuli such as others’ visual or verbal reactions are minimized (Ando and Sakamoto, 2008). As a result, individuals with psychosocial problems find it easier to present themselves on social networks (Chen et al., 2024). In offline interactions, they often anticipate negative evaluations and rejection, leading them to immerse themselves in social networks to avoid such scenarios (Di Blasi et al., 2015; Shensa et al., 2017). Research indicates that adolescents with psychosocial issues prefer online social interactions over face-to-face communication because social networks compensate for their deficiencies in social skills (Lee-Won et al., 2015; O’Day and Heimberg, 2021). This preference for online interactions can lead to compulsive social network engagement, ultimately resulting in social network site addiction (Caplan, 2003). The I-PACE model identifies psychosocial issues as significant underlying factors contributing to social network site addiction (Brand et al., 2019). Based on the above review, this study hypothesizes that depression, anxiety, and stress mediate the relationship between bullying victimization and adolescents’ social network site addiction.

Prior research has suggested that bullying victimization can impair self-regulatory processes, including inhibitory control, which may increase the likelihood of problematic behaviors such as social network site addiction. However, the specific mediating role of inhibitory control in this pathway remains underexplored, particularly among adolescents. This study addresses this gap by hypothesizing and testing whether inhibitory control mediates the relationship between bullying victimization and social network site addiction. By focusing on this mechanism, the study contributes to a deeper understanding of the psychological pathways linking victimization and addiction, offering novel insights into the developmental vulnerabilities of adolescents. As a higher-order cognitive function, inhibitory control refers to the ability to suppress interfering responses and attention tendencies to achieve goal-directed tasks (Miyake and Friedman, 2012). Inhibitory control plays a critical role in adolescents’ learning and daily life, with early adolescence being a key period for its rapid development (Brocki and Bohlin, 2004). Impairments in inhibitory control can lead to issues such as internet addiction (Dong et al., 2012; Koob and Volkow, 2010; Liu et al., 2024a,b,c,d,e), impulsive decision-making or behavior (Mirabella, 2021), and poor management of negative emotions (Chiang et al., 2024). Previous research has shown a strong connection between bullying victimization and inhibitory control (Edalati et al., 2018; Liu et al., 2024a,b,c,d,e), with bullying victimization impairing executive and cognitive functions (Medeiros et al., 2016). Experimental studies have found abnormal activation in brain regions involved in inhibitory control (such as the prefrontal cortex, caudate nucleus, and subthalamic nucleus) among bullying victims (Palamarchuk and Vaillancourt, 2022). Longitudinal studies indicate that bullying victimization significantly predicts a decline in inhibitory control, and the two are negatively correlated (Hogye et al., 2022; Reijntjes et al., 2010; Liu et al., 2024a,b,c,d,e). Social information processing theory posits that individuals who experience bullying victimization may develop negative social cognitive patterns, leading to incorrect attributions or hypersensitivity in social situations, thereby impairing their emotional regulation and inhibitory control (Zeytinoglu et al., 2023). Moreover, research suggests that impaired inhibitory control is a risk factor for various forms of internet addiction (Antons and Matthias, 2020; Liu et al., 2024a,b,c,d,e), with lower inhibitory control predicting increased gaming time in individuals with internet gaming disorder (IGD) (Kräplin et al., 2020). Adolescents with social network site addiction also exhibit lower levels of inhibitory control (Dieter et al., 2017). The I-PACE model hypothesizes that the development of social network site addiction results from the interaction between individual vulnerability variables, emotional and cognitive responses to specific stimuli, and impairments in executive functions, such as inhibitory control and decision-making (Brand et al., 2019). Lower inhibitory control is believed to contribute to the development and maintenance of social network site addiction. Generally reduced inhibitory control may increase the risk of addiction and its recurrence, as individuals struggle to adequately regulate attention, emotions, and behavior in pursuit of long-term goals, while their ability to inhibit habit-driven, impulsive, and reward-seeking responses triggered by cues is compromised (Dieter et al., 2017). Based on this review, this study hypothesizes that inhibitory control mediates the relationship between bullying victimization and adolescents’ social network site addiction.

Increasing evidence also supports the close relationship between depression, anxiety, stress, and inhibitory control. Research has shown that individuals with more severe depressive symptoms not only exhibit poorer inhibitory control but are also more prone to impulsive behaviors during tasks, making it difficult to resist immediate temptations, leading to behavioral fluctuations (Nahum et al., 2023). Individuals with anxiety-related disorders typically demonstrate deficits in inhibitory control (Mclaughlin et al., 2016). In anxious states, individuals remain overly alert to potential threats, resulting in cognitive overload, which diminishes their capacity to inhibit impulsive behaviors (Wang et al., 2024).

According to attentional control theory, anxiety impairs executive attention functions, particularly inhibitory control (Eysenck and Derakshan, 2011). Additionally, research has shown that individuals under stress exhibit an increased tendency toward immediate gratification, weakening their focus on long-term goals and thereby impairing inhibitory control (Roos et al., 2017). Based on the above discussion, this study hypothesizes that depression, anxiety, and stress are negatively correlated with inhibitory control, and depression, anxiety, stress, and inhibitory control play a chain mediating role between bullying victimization and adolescent social network site addiction.

**Figure 1:**
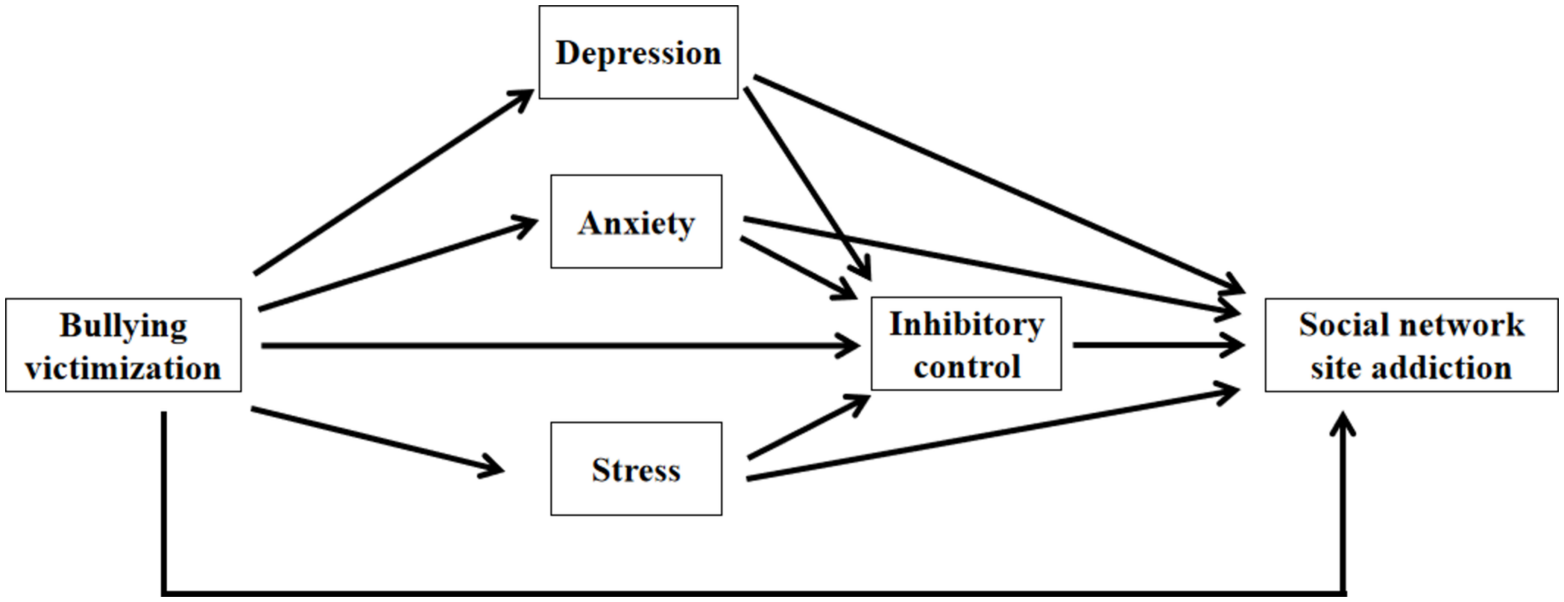
Hypothesized a mediation model.

In conclusion, previous studies have explored the relationship and predictive role between bullying victimization and social network addiction, while research on the negative emotions and inhibitory control factors involved in this relationship remains limited. To further fill this gap and explore the underlying psychological mechanisms, this study introduces depression, anxiety, stress, and inhibitory control as mediating variables. Based on this, the study proposes the following hypotheses and constructs a hypothesized path model (Figure 1).

Hypothesis 1: Bullying victimization significantly predicts the occurrence of social network site addiction among adolescents.

Hypothesis 2a: Depression mediate the relationship between bullying victimization and adolescents’ social network site addiction.

Hypothesis 2b: Anxiety mediate the relationship between bullying victimization and adolescents’ social network site addiction.

Hypothesis 2c: Stress mediate the relationship between bullying victimization and adolescents’ social network site addiction.

Hypothesis 3: Inhibitory control mediates the relationship between bullying victimization and adolescents’ social network site addiction.

Hypothesis 4a: Depression and inhibitory control play a chain mediating role between bullying victimization and adolescent social network site addiction.

Hypothesis 4b: Anxiety and inhibitory control play a chain mediating role between bullying victimization and adolescent social network site addiction.

Hypothesis 4c: Stress and inhibitory control play a chain mediating role between bullying victimization and adolescent social network site addiction.

## 2 Methods

### 2.1 Participants

The present study was conducted during the fall semester of 2023, utilizing convenience sampling to recruit 1,015 middle school students from six schools located in Shandong, Hebei, Henan, and Hunan provinces in China. Prior to the survey, ethical approval was obtained from the university’s institutional ethics committee to ensure compliance with ethical research standards. The survey was administered using an online electronic questionnaire, which was distributed in a class-based format. To ensure consistency in data collection procedures, all research staff involved in the survey received standardized training. Teachers from each class facilitated the distribution and completion of the questionnaires among their students, with support from the research team. Before administering the questionnaire, the teachers explained the purpose of the study to the students and clarified the anonymous and confidential nature of the data, the intended use of the data, and the voluntary nature of participation. Participants were also informed of their right to withdraw from the study at any time without any consequences.

The completion of the questionnaire required no more than 20 min. To ensure data quality, a reasonable minimum response time was established based on the questionnaire’s length and complexity, and submissions with response times below this threshold were deemed invalid. Written informed consent was obtained from all participants and their legal guardians before participation. Guardians were provided with clear information regarding the study objectives, the nature of the data collected, and measures to ensure confidentiality and safety. After data collection, the responses were carefully screened to exclude incomplete submissions and data with obvious response patterns.

A total of 1,015 questionnaires were distributed, and 1,005 were deemed valid after excluding invalid responses, resulting in a high effective response rate of 99.01%. The final analysis sample included 1,005 participants, comprising 488 boys and 517 girls, with 177 identifying as only children and 828 as non-only children. The average age of the participants was 13.87 years (SD = 1.62). These rigorous data collection and screening procedures ensured the reliability and validity of the dataset for subsequent analysis.

### 2.2 Measures

#### 2.2.1 Bullying victimization

Bullying victimization was assessed using a single question that provided a detailed definition of bullying, describing it as “repeated and frequent negative actions inflicted by others in a situation of unequal power or status, such as hitting, kicking, shoving, threatening, mocking, insulting, excluding, spreading rumors, or sending hurtful emails or messages” (Olweus, 1993). Participants were asked to recall their experiences in the past 30 days and respond using a 6-point scale (0 = never, 1 = once a month, 2 = two to three times a month, 3 = once a week, 4 = several times a week, 5 = almost every day). This tool has been utilized in prior studies (Badarch et al., 2022; Gredler, 2003; Hosozawa et al., 2021; Khan et al., 2020; Koyanagi et al., 2019; Koyanagi et al., 2020; Liu et al., 2024a,b,c,d,e; Smith et al., 2021; Waasdorp et al., 2019; Yang Y. X. et al., 2023; Yang W. Y. et al., 2023).

#### 2.2.2 Social network site addiction

Social network site addiction was assessed using a modified version of the scale developed by Elphinston and Noller (2011), which was adapted, revised, and validated by Wei (2018) for measuring addiction to social network sites among adolescents. The scale includes eight items, covering aspects such as feelings and frequency of social network use, impacts on daily life (e.g., study, social activities, sleep), and withdrawal symptoms. Each item was rated on a 5-point Likert scale ranging from 1 (completely disagree) to 5 (completely agree). The total score, ranging from 8 to 40, represented the level of social network site addiction, with higher scores indicating more severe addiction. The Cronbach’s *α* for the sample in this study was 0.845. The KMO value of the scale was 0.888, and *p* < 0.001, indicating good construct validity.

#### 2.2.3 Depression, anxiety, and stress

The levels of depression, anxiety, and stress were measured using the Depression, Anxiety, and Stress Scale (DASS-21) developed by Lovibond and Lovibond (1995) and validated in China by Gong et al. (2010). This 21-item scale uses a 4-point Likert scale (1 = does not apply, 4 = applies very much) to measure the severity of depression, anxiety, and stress, with total scores ranging from 21 to 84. Higher scores indicate higher levels of these mental health issues. The Cronbach’s α for the sample in this study ranged from 0.797 to 0.863. The KMO values of the scale ranged from 0.863 to 0.883, and *p* < 0.001, indicating good construct validity.

#### 2.2.4 Inhibitory control

The inhibitory control subscale from the Executive Function Scale developed by Huang et al. (2014) was employed to measure the inhibitory control level of adolescents. The subscale included 6 items, each rated from 1 (often) to 3 (never). The total score of the items represented the level of inhibitory control, ranging from 6 to 18, with higher scores indicating higher levels of inhibitory control. The Cronbach’s α for the sample in this study was 0.791. The KMO value of the scale was 0.818, and *p* < 0.001, indicating good construct validity.

### 2.3 Data processing and analysis

Data entry was performed using Excel 2021. Normality tests were then conducted using SPSS 26.0, revealing that the variables of bullying victimization, social network site addiction, depression, anxiety, stress, and inhibitory control all followed a normal distribution. For variables that met the assumption of normality, descriptive statistics were calculated using means (M) and standard deviations (SD). A method bias test was conducted, with a threshold of 50% indicating no significant common method bias (Podsakoff et al., 2003). Pearson correlation analysis was then used to assess the relationships between the main variables. Prior to further analysis, the data for the main variables were standardized. To test our hypotheses, the PROCESS plugin (Model 80) in SPSS was used to examine the relationship between bullying victimization and social network site addiction, and to explore the mediating effects of depression, anxiety, stress, and inhibitory control (Hayes, 2017). Bootstrapping with 5,000 iterations was used to assess model fit and estimate 95% confidence intervals (95% CI), ensuring robust data analysis (Berkovits et al., 2000). Gender and grade were controlled as covariates throughout the analysis. The significance level was set at 0.05.

## 3 Results

### 3.1 Common method bias test

To assess the impact of common method bias, we applied Harman’s single-factor test. The analysis revealed two factors with eigenvalues greater than 1 before the rotation of principal components. The first factor explained 41.96% of the variance, which is below the 50% threshold. Therefore, there is no significant common method bias present in this study.

### 3.2 Descriptive analysis

Table 1 presents the differences in key variables between boys and girls and between only children and non-only children. Significant differences were observed between boys and girls in inhibitory control (*t* = 2.04, *p* < 0.05), social network site addiction (*t* = −5.78, *p* < 0.001), depression (*t* = −5.07, *p* < 0.001), anxiety (*t* = −5.88, *p* < 0.001), and stress (*t* = −6.48, *p* < 0.001). Boys scored lower than girls in social network site addiction, depression, anxiety, and stress, while girls scored lower than boys in inhibitory control. No significant differences were found between only children and non-only children across bullying victimization, inhibitory control, social network site addiction, depression, anxiety, or stress.

**Table 1:** Describes the analysis.

Variables		Bullying victimization		Inhibitory control		Social network site addiction		Depression		Anxiety		Stress	
		Mean	SD	Mean	SD	Mean	SD	Mean	SD	Mean	SD	Mean	SD
Gender	Boys	0.35	0.78	14.19	2.53	17.36	6.92	11.63	4.36	12.57	4.27	12.64	4.31
	Girls	0.42	0.79	13.87	2.55	19.97	7.35	13.08	4.69	14.18	4.39	14.44	4.49
	*t*	−1.44		2.04*		−5.78***		−5.07***		−5.88***		−6.48***	
Only child status	Only child	0.47	0.82	14.06	2.55	17.79	7.15	12.44	4.89	13.19	4.47	13.00	4.46
	Non-only child	0.37	0.78	14.02	2.54	18.90	7.27	12.36	4.53	13.45	4.40	13.69	4.49
	*t*	1.60		0.17		−1.88		0.20		−0.71		−1.86	

*: *p*<0.05; ***: *p*<0.001.

### 3.3 Correlation analysis between variables

As shown in Table 2, bullying victimization was negatively correlated with adolescents’ inhibitory control (*r* = −0.24, *p* < 0.001) and positively correlated with social network site addiction (*r* = 0.13, *p* < 0.001), depression (*r* = 0.24, *p* < 0.001), anxiety (*r* = 0.27, *p* < 0.001), and stress (*r* = 0.24, *p* < 0.001). Social network site addiction was significantly negatively correlated with inhibitory control (*r* = −0.29, *p* < 0.001) and positively correlated with depression (*r* = 0.41, *p* < 0.001), anxiety (*r* = 0.43, *p* < 0.001), and stress (*r* = 0.46, *p* < 0.001). Additionally, inhibitory control was significantly negatively correlated with depression (*r* = −0.41, *p* < 0.001), anxiety (*r* = −0.45, *p* < 0.001), and stress (*r* = −0.47, *p* < 0.001). Finally, depression, anxiety, and stress were all positively correlated with each other.

**Table 2:** Correlation analysis between variables.

	1	2	3	4	5	6
1 Age	-					
2 Bullying victimization	−0.13***	-				
3 Social network site addiction	0.16***	0.13***	-			
4 Inhibitory control	0.09**	−0.24***	−0.29***	-		
5 Depression	0.17***	0.24***	0.41***	−0.41***	-	
6 Anxiety	0.03	0.27***	0.43***	−0.45***	0.75***	-
7 Stress	0.11***	0.24***	0.46***	−0.47***	0.76***	0.84***

**: *p*<0.01; ***: *p*<0.001.

### 3.4 Mediator model checking

**Figure 2:**
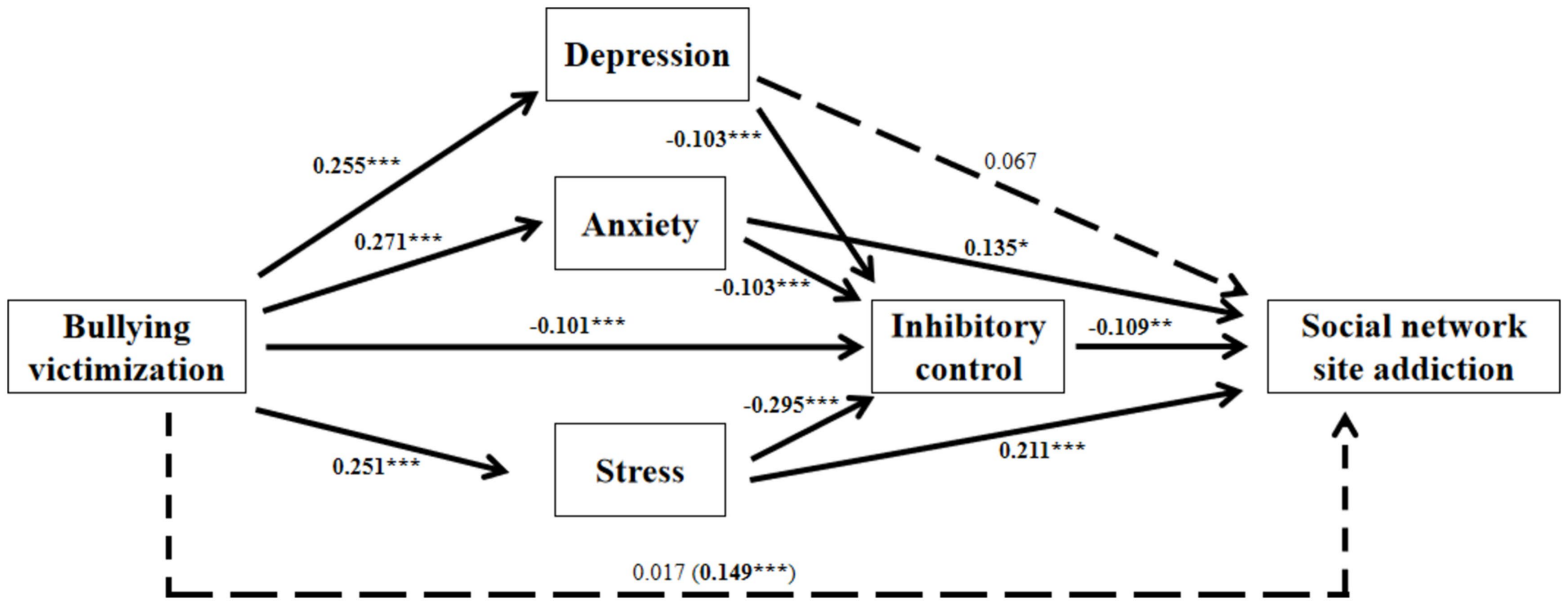
Model with inhibitory control as the central mediator.

After controlling for gender and age, bullying victimization was found to significantly and positively predict adolescents’ social network site addiction (β = 0.149, SE = 0.031, *p* < 0.001). However, when analyzing the indirect effects, bullying victimization no longer significantly predicted social network site addiction (β = 0.017, SE = 0.029, *p* > 0.05). Bullying victimization significantly and positively predicted depression (β = 0.255, SE = 0.030, *p* < 0.001), anxiety (β = 0.271, SE = 0.030, *p* < 0.001), and stress (β = 0.251, SE = 0.030, *p* < 0.001). Additionally, bullying victimization (β = −0.101, SE = 0.029, *p* < 0.001), depression (β = −0.103, SE = 0.046, *p* < 0.05), anxiety (β = −0.113, SE = 0.054, *p* < 0.05), and stress (β = −0.295, SE = 0.055, *p* < 0.001) significantly and negatively predicted adolescents’ inhibitory control. Anxiety (β = 0.135, SE = 0.055, *p* < 0.05) and stress (β = 0.211, SE = 0.056, *p* < 0.001) both significantly and positively predicted adolescents’ social network site addiction. Lastly, inhibitory control negatively predicted social network site addiction (β = −0.109, SE = 0.032, *p* < 0.01) (see Figure 2; Tables 3, 4).

**Table 3:** Test of chain mediation model.

Outcome variable	Predictor variable	β	SE	*t*	R^2^	*F*
Social network site addiction	Bullying victimization	0.149	0.031	4.871***	0.081	29.416***
	Age	0.176	0.030	5.810***		
	Gender	0.184	0.031	6.030***		
Depression	Bullying victimization	0.255	0.030	8.627***	0.121	45.873***
	Age	0.149	0.029	5.073***		
	Gender	0.207	0.030	6.992***		
Anxiety	Bullying victimization	0.271	0.030	9.100***	0.108	40.465***
	Age	0.170	0.030	5.738***		
	Gender	0.068	0.030	2.294*		
Stress	Bullying victimization	0.251	0.030	8.432***	0.117	44.058***
	Age	0.191	0.030	6.452***		
	Gender	0.150	0.030	5.035***		
Inhibitory control	Bullying victimization	−0.101	0.029	−3.522***	0.261	58.710***
	Depression	−0.103	0.046	−2.249*		
	Anxiety	−0.113	0.054	−2.704*		
	Stress	−0.295	0.055	−5.380***		
	Age	0.038	0.028	1.363		
	Gender	0.129	0.028	4.528***		
Social network site addiction	Bullying victimization	0.017	0.029	0.592	0.255	48.799***
	Depression	0.067	0.046	1.459		
	Anxiety	0.135	0.055	2.462*		
	Stress	0.211	0.056	3.778***		
	Inhibitory control	−0.109	0.032	−3.431**		
	Age	0.098	0.028	3.488**		
	Gender	0.136	0.029	4.700***		

*: *p*<0.05; **: *p*<0.01; ***: *p*<0.001.

**Table 4:** Inhibitory control as the central mediating effect.

Intermediate path	Effect size	SE	Bootstrap 95% CI	Proportion of mediating effect
Total effect	0.149	0.031	0.089, 0.209	
Direct effect	0.017	0.029	−0.040, 0.074	
Total indirect effect	0.132	0.018	0.100, 0.169	88.59%
Bullying victimization→Depression→Social network site addiction	0.017	0.013	−0.007, 0.045	11.41%
Bullying victimization→Anxiety→Social network site addiction	0.036	0.016	0.007, 0.069	24.16%
Bullying victimization→Stress→Social network site addiction	0.053	0.016	0.024, 0.087	35.57%
Bullying victimization→Inhibitory control→Social network site addiction	0.011	0.005	0.003, 0.022	7.38%
Bullying victimization→Depression→Inhibitory control→Social network site addiction	0.003	0.002	0.001, 0.007	2.01%
Bullying victimization→Anxiety→Inhibitory control→Social network site addiction	0.003	0.002	0.001, 0.008	2.01%
Bullying victimization→Stress→Inhibitory control→Social network site addiction	0.008	0.003	0.003, 0.015	5.37%

## 4 Discussion

This study explored the relationships between bullying victimization, social network site addiction, depression, anxiety, stress, and inhibitory control. Additionally, we examined the mediating roles of depression, anxiety, stress, and inhibitory control within this model. Our findings revealed a significant positive correlation between bullying victimization and adolescents’ social network site addiction. However, when negative emotions (depression, anxiety, and stress) and inhibitory control were introduced as mediating variables, this relationship was no longer significant. Specifically, bullying victimization significantly predicted negative emotions and was negatively associated with inhibitory control. Negative emotions were significantly negatively related to inhibitory control, and inhibitory control, in turn, negatively predicted social network site addiction. Among the negative emotions, stress and anxiety were significantly positively associated with social network site addiction, validating our initial hypothesis.

Our study found a significant positive correlation between bullying victimization and adolescents’ social network site addiction. However, we further demonstrated that when negative emotions (such as depression, anxiety, and stress) and inhibitory control were added as mediating variables, the direct association between bullying victimization and social network site addiction was no longer significant. This suggests that the mediating variables play a crucial role, diluting the direct effect of bullying victimization on social network site addiction or influencing addiction behaviors indirectly through other psychological mechanisms. Bullying victimization refers to the repeated intentional harm inflicted by one or more students over time (Mcrae and Gross, 2020), a high-prevalence experience for adolescents (10–35%) (Moore et al., 2017). The results of our study can be explained through the self-medication hypothesis, which suggests that social network site addiction may serve as a coping strategy to alleviate negative emotions triggered by traumatic experiences or life stress (Hsieh et al., 2016). Individuals who have experienced stressful events often tend to rely on substances or behaviors to avoid adverse outcomes (Greeley and Oei, 1999). In the context of bullying victimization, individuals may immerse themselves in social network activities to escape repeated victimization and cope with the negative effects of being bullied (Zhai et al., 2019). Empirical research supports this view, showing that bullying victimization can lead to addictive online behaviors, allowing individuals to distance themselves from distress and negative emotions following exposure to stressful events (Zhao et al., 2020). Consequently, social network site addiction can be regarded as a maladaptive coping strategy similar to substance use. Although this behavior may temporarily relieve the emotional distress caused by victimization, it is an unhealthy coping mechanism, reflecting a dysregulated response to traumatic experiences. Moreover, the compensatory satisfaction theory suggests that when individuals struggle to meet their expectations in real life, they may turn to alternative means to seek fulfillment (Lu et al., 2020). This may be particularly relevant in the Chinese context, where collectivist values and high parental expectations often exacerbate stress levels in adolescents (Wang et al., 2017). In this context, social network activities provide an alternative platform for obtaining attention and positive feedback from others, partially compensating for the lack of social connections in real life. This behavior not only fulfills their need for social validation but also helps alleviate the negative emotions associated with bullying victimization (Lu et al., 2020). This study contributes by emphasizing the cultural and social factors that uniquely shape these relationships, offering a perspective distinct from prior studies conducted in Western contexts. In summary, our findings demonstrate a positive correlation between bullying victimization and Chinese adolescents’ social network site addiction (H1).

Our study provides new insights into the mediating role of anxiety and stress in the relationship between bullying victimization and adolescents’ social network site addiction, highlighting the psychological mechanisms underlying this association. While prior research has established strong relationships between bullying victimization and negative emotions, as well as between negative emotions and social network site addiction (Balluerka et al., 2023; Lee, 2021; Li et al., 2022; Yang Y. X. et al., 2023; Yang W. Y. et al., 2023), our findings expand this understanding by contextualizing these relationships within a mediational framework specific to Chinese adolescents. This cultural context is particularly relevant, as China’s collectivist values, intense academic pressures, and unique social media environment shape adolescents’ emotional responses and online behaviors in ways that differ significantly from Western contexts (Hu et al., 2024). Adolescents who experience bullying victimization are often isolated from or intentionally distanced by their peers, leading to internalizing problems such as depression, anxiety, and stress (Reijntjes et al., 2010). Adolescents who suffer from anxiety and stress are often introverted and reluctant to communicate with peers, but they may turn to social networks to escape or eliminate these negative emotions, ultimately leading to excessive reliance on the internet (Liu et al., 2022). The social compensation theory (Valkenburg and Peter, 2009) further explains that individuals experiencing negative emotions are more likely to seek support in the virtual world, using social networks to mitigate negative emotions and/or cope with stress or anxiety caused by functional impairments in their real lives. Those suffering from anxiety and stress may overuse social networks as a means of changing their circumstances, potentially experiencing conflicts with real-world obligations and desires due to this excessive use. These conflicts may exacerbate their depression, anxiety, and stress, and when they fail to reduce their social network usage, further emotional distress may ensue. As emotional shifts, stress, and anxiety intensify, the likelihood of social network site addiction increases. This explanation is supported by previous cross-sectional and longitudinal studies in addiction psychology (Chang et al., 2022; Chen et al., 2020). Our study further indicates that among the dimensions of negative emotions, stress has the most significant predictive effect on social network site addiction. This may be due to the interaction of multiple factors. Adolescents face a broad range of stressors, including academic pressure, interpersonal tensions, family expectations and responsibilities, a lack of social acceptance, and adaptive challenges posed by rapidly changing social environments (Compas et al., 2001). The high expectations parents and society place on adolescents’ academic performance and future achievements often exceed their psychological capacity, prompting them to seek ways to escape the pressures of reality. Due to its instant feedback mechanisms and virtualized social environment, social networks have become a primary means of stress relief for adolescents. However, excessive reliance on this coping mechanism can lead to social network site addiction, exacerbating the psychological and behavioral consequences and increasing the risk of addiction (Ghobadzadeh et al., 2019). In summary, our study demonstrated that anxiety and stress mediate the relationship between bullying victimization and social network site addiction in adolescents (H2b and H2c).

This study found that inhibitory control plays a mediating role between bullying victimization and adolescents’ social network site addiction, supporting our initial hypothesis (H3). Victims of bullying may experience negative emotions or psychological discomfort due to repeated attacks. This not only reduces their opportunities for social interaction but also depletes cognitive resources, weakening their ability to self-regulate impulsive and reward-seeking behaviors. Inhibitory control influences how adolescents manage impulsive behavior and cope with negative emotions, shaping their online behavior. When inhibitory control is weakened, individuals may turn to social networks to escape negative emotions, rather than engaging in emotional regulation or seeking appropriate support. Our results showed a negative correlation between bullying victimization and inhibitory control. It is well-established that the prefrontal cortex and dopamine reward pathways regulate inhibitory control cognition (Diamond, 2013). Chronic stress caused by bullying victimization may activate the hypothalamic–pituitary–adrenal (HPA) axis, leading to elevated cortisol levels. This in turn can impair prefrontal cortex function, reducing impulse control and self-regulation abilities (Menken et al., 2023). Chronic stress from bullying may also affect the dopamine signaling pathways of the mesolimbic system, which is involved in reward and motivation. Dysregulation of the dopamine system can lead to increased impulsive behavior and over-reinforced reward-seeking, further weakening inhibitory control (Palamarchuk and Vaillancourt, 2022). Positive peer relationships, which are critical for adolescents, can help develop higher levels of inhibitory control (Oberle and Schonert-Reichl, 2013). Increasing evidence suggests that social network site addiction may result from inhibitory control deficits and impaired executive control networks in the frontal lobes (Wegmann et al., 2020). Our findings support this view. Behavioral and neurophysiological studies also provide evidence that deficits in inhibitory control contribute to addictive behaviors related to social network site use (Ko et al., 2014; van den Heuvel and Hulshoff Pol, 2010). Furthermore, according to the ego depletion theory, emotional regulation consumes cognitive resources. When these resources are depleted over time, inhibitory control declines, increasing the likelihood of risk behaviors and social network site addiction (Evans et al., 2016).

Additionally, this study found a negative correlation between depression, anxiety, stress, and inhibitory control. The negative correlation between depression and inhibitory control may be attributed to impaired function in the prefrontal cortex, a key region for executive functions such as emotional regulation, cognitive control, and impulse inhibition. When individuals are in a depressive state, they become more sensitive to negative stimuli in their environment, leading to cognitive resources being consumed by emotional processing, which weakens inhibitory control (Nahum et al., 2023). ERP studies have shown that highly anxious individuals may exhibit delayed responses during inhibitory tasks, with reduced brain activity in areas associated with the inhibition process (Ansari and Derakshan, 2011). This may be because anxiety directly impacts the prefrontal cortex, affecting functions such as working memory and cognitive execution (Arnsten and Rubia, 2012). When individuals face social stress, particularly social exclusion or bullying, the brain’s emotional regulation regions (such as the amygdala and cingulate gyrus) are activated, while the prefrontal cortex’s ability to regulate these emotional responses is diminished. Under prolonged social stress, the brain becomes hypersensitive to negative social cues, increasing emotional reactivity and neglecting long-term goals, which further reduces inhibitory control (Liu et al., 2021). Based on the above, depression, anxiety, and stress are negatively correlated with inhibitory control. Furthermore, anxiety, stress, and inhibitory control play a chain mediation role between bullying victimization and adolescent social network site addiction, thereby confirming our final hypothesis (H4b and H4c).

This study examined the mediating roles of negative emotions and inhibitory control in the relationship between bullying victimization and social network site addiction among Chinese adolescents, thereby extending the applicability of these findings to collectivist cultural contexts. It underscores how adolescents’ psychological and behavioral responses to bullying are influenced by China’s unique social context, which places significant emphasis on academic achievement and family obligations. By incorporating cultural dimensions into the analysis of these variables, this study enhances our understanding of how environmental factors shape pathways leading to social network site addiction. The findings suggest that emotion dysregulation and cognitive impairments work in tandem to promote maladaptive online behaviors, offering a framework for future research on these interactions in different cultural and developmental contexts. The study also has important practical implications. Interventions aimed at reducing social network site addiction should prioritize strengthening inhibitory control and addressing the emotional consequences of bullying victimization. Schools and mental health practitioners in China should focus on programs designed to improve adolescents’ emotion regulation and cognitive resilience, particularly in light of the unique stressors present in this cultural context. Additionally, family and school-based interventions should work to mitigate the impact of academic and social stress on adolescents, offering alternative coping mechanisms to reduce their reliance on digital platforms for emotional relief.

This study has several limitations. First, the use of self-report survey data may introduce bias, such as inaccuracies resulting from subjective perceptions or memory recall errors. Second, the sample lacks sufficient diversity, which may limit the generalizability of the findings and reduce external validity. Third, the cross-sectional design of the study prevents the establishment of causal relationships. Fourth, the scope of the demographic characteristics collected could be expanded in future research. Future studies should consider adopting longitudinal designs to more robustly explore causal relationships. Experimental methods could also be employed to validate the mediating effects identified in this study. Additionally, research should further investigate the mechanisms through which environmental factors, such as family dynamics and academic pressure, influence adolescents’ coping strategies and online behaviors. Longitudinal studies would be particularly valuable in examining how these factors evolve over time and their long-term impact on adolescents’ well-being. Furthermore, studies assessing the effectiveness of targeted interventions, such as cognitive-behavioral therapy and emotion regulation programs, are critical for developing evidence-based strategies to mitigate social network site addiction.

## 5 Conclusion

This study explored the relationships between bullying victimization, adolescents’ social network site addiction, depression, anxiety, stress, and inhibitory control. It constructed a model where depression, anxiety, stress, and inhibitory control mediate the link between bullying victimization and social network site addiction. It is critical for individuals, families, schools, and society to acknowledge the negative consequences of social network site addiction. Focus should be placed on stratified and specific assessments based on measurable results, which can guide the development of targeted and personalized intervention strategies.

## Data Availability

The raw data supporting the conclusions of this article will be made available by the authors, without undue reservation.
