# Supplementary material for: The central mediating effect of inhibitory control and negative emotion on the relationship between bullying victimization and social network site addiction in adolescents
Source: Front Psychol. 2025 Apr 2;15:1520404. doi: 10.3389/fpsyg.2024.1520404 (PMC12002087; doi:10.3389/fpsyg.2024.1520404)
Supplement: Supplementary file 1 [file Data_Sheet_1.docx]

Measures

2 Social network site addiction

| 1 = Not at all true, 2 = Not true, 3 = Not sure, 4 = True, 5 = Completely true. | | | | | | |
| --- | --- | --- | --- | --- | --- | --- |
| NO. | Item | 1 | 2 | 3 | 4 | 5 |
| 1 | Using social networking sites makes it difficult for me to concentrate on my studie. |  |  |  |  |  |
| 2 | The first thing I think of when I wake up every morning is logging into social networking sites. |  |  |  |  |  |
| 3 | Excessive use of social networking sites makes it difficult for me to fall asleep. |  |  |  |  |  |
| 4 | Using social networking sites interferes with my daily social activities. |  |  |  |  |  |
| 5 | My family and friends think I spend too much time on social networking sites. |  |  |  |  |  |
| 6 | Not being able to use social networking sites makes me feel restless. |  |  |  |  |  |
| 7 | When I feel down, I often log into social networking sites to make myself feel better. |  |  |  |  |  |
| 8 | I have tried to reduce the time I spend on social networking sites, but I ultimately failed. |  |  |  |  |  |

3 Depression, anxiety and stress

| 1 = Not true at all, 2 = Somewhat untrue, 3 = Somewhat true, 4 = Completely true. | | | | | |
| --- | --- | --- | --- | --- | --- |
| NO. | Item | 1 | 2 | 3 | 4 |
| 1 | I find it difficult to calm myself down. |  |  |  |  |
| 2 | I feel dry mouth and thirst. |  |  |  |  |
| 3 | I am unable to feel truly positive or optimistic. |  |  |  |  |
| 4 | I experience difficulty breathing, even without physical exertion, or feel short of breath. |  |  |  |  |
| 5 | I find it very difficult to take the initiative to start studying. |  |  |  |  |
| 6 | I often react with excessive sensitivity to situations. |  |  |  |  |
| 7 | I have experienced trembling (e.g., hand shaking). |  |  |  |  |
| 8 | I frequently feel nervous or tense. |  |  |  |  |
| 9 | I worry about situations that may cause panic or embarrassment. |  |  |  |  |
| 10 | I feel there is little to look forward to in the future. |  |  |  |  |
| 11 | I feel uneasy and restless. |  |  |  |  |
| 12 | I find it very difficult to relax. |  |  |  |  |
| 13 | I feel sad and depressed. |  |  |  |  |
| 14 | I cannot tolerate anything that prevents me from continuing my studies. |  |  |  |  |
| 15 | I feel like I am losing control. |  |  |  |  |
| 16 | I am unable to feel enthusiasm for anything. |  |  |  |  |
| 17 | I feel that I have no value or purpose in life. |  |  |  |  |
| 18 | I find myself easily angered. |  |  |  |  |
| 19 | Even without obvious physical activity, I experience a rapid heartbeat or irregular heartbeat. |  |  |  |  |
| 20 | I feel fearful for no apparent reason. |  |  |  |  |
| 21 | I feel that life has no meaning. |  |  |  |  |

4 Inhibitory control

| 1 = Frequently; 2 = Sometimes; 3 = Never. | | | | |
| --- | --- | --- | --- | --- |
| NO. | Item | 1 | 2 | 3 |
| 1 | I am impulsive. |  |  |  |
| 2 | I overreact. |  |  |  |
| 3 | I speak at inappropriate times. |  |  |  |
| 4 | I act without considering the consequences. |  |  |  |
| 5 | I speak without thinking. |  |  |  |
| 6 | My behavior is too wild or "out of control." |  |  |  |
